# S100A8/S100A9 Promote Progression of Multiple Myeloma via Expansion of Megakaryocytes

**DOI:** 10.1158/2767-9764.CRC-22-0368

**Published:** 2023-03-13

**Authors:** Cindy Lin, Laura Garcia-Gerique, Erin E. Bonner, Jerome Mastio, Matthew Rosenwasser, Zachary Cruz, Michael Lawler, Luca Bernabei, Kar Muthumani, Qin Liu, Mortimer Poncz, Thomas Vogl, Marie Törngren, Helena Eriksson, Dan T. Vogl, Dmitry I. Gabrilovich, Yulia Nefedova

**Affiliations:** 1 The Wistar Institute, Philadelphia, Pennsylvania.; 2 ICC, Early Oncology R&D, AstraZeneca, Cambridge, United Kingdom.; 3 Abramson Cancer Center, University of Pennsylvania, Philadelphia, Pennsylvania.; 4 GeneOne Life Science, Inc, Fort Washington, Pennsylvania.; 5 Children's Hospital of Philadelphia, Philadelphia, Pennsylvania.; 6 University of Münster, Münster, Germany.; 7 Active Biotech AB, Lund, Sweden.; 8 ICC, Early Oncology R&D, AstraZeneca, Gaithersburg, Maryland.

## Abstract

Multiple myeloma is characterized by clonal proliferation of plasma cells that accumulate preferentially in the bone marrow (BM). The tumor microenvironment is one of the leading factors that promote tumor progression. Neutrophils and monocytes are a major part of the BM tumor microenvironment, but the mechanism of their contribution to multiple myeloma progression remains unclear. Here, we describe a novel mechanism by which S100A8/S100A9 proteins produced by BM neutrophils and monocytes promote the expansion of megakaryocytes supporting multiple myeloma progression. S100A8/S100A9 alone was not sufficient to drive megakaryopoiesis but markedly enhanced the effect of thrombopoietin, an effect that was mediated by Toll-like receptor 4 and activation of the STAT5 transcription factor. Targeting S100A9 with tasquinimod as a single agent and in combination with lenalidomide and with proteasome inhibitors has potent antimyeloma effect that is at least partly independent of the adaptive immune system. This newly identified axis of signaling involving myeloid cells and megakaryocytes may provide a new avenue for therapeutic targeting in multiple myeloma.

**Significance:**

We identified a novel mechanism by which myeloid cells promote myeloma progression independently of the adaptive immune system. Specifically, we discovered a novel role of S100A8/S100A9, the most abundant proteins produced by neutrophils and monocytes, in regulation of myeloma progression via promotion of the megakaryocyte expansion and angiogenesis. Tasquinimod, an inhibitor of S100A9, has potent antimyeloma effects as a single agent and in combination with lenalidomide and with proteasome inhibitors.

## Introduction

Multiple myeloma is an incurable blood cancer characterized by clonal proliferation of plasma cells that accumulate preferentially in the bone marrow (BM). The tumor microenvironment (TME) is one of the leading factors that promote tumor progression. We and others have previously demonstrated that multiple myeloma progression is associated with accumulation in the BM of myeloid-derived suppressor cells (MDSC; refs. 1–5). These cells are morphologically and phenotypically similar to neutrophils or monocytes but exist in a state of pathologic activation characterized by distinct functional and biochemical features and the ability to suppress antitumor immune responses (6). The specific contribution of these cells to multiple myeloma progression and possible approaches to their therapeutic targeting remain largely unclear. One of the main features of myeloid cells, including neutrophils, monocytes, as well as MDSC, is high expression and release of S100A8 and S100A9 proteins. These Ca^2+^ and Zn^2+^ binding proteins are produced and released by neutrophils and monocytes and typically form stable heterodimers *in vivo* (7). Intracellular S100A8/S100A9 participate in cytoskeleton modulation, arachidonic acid (AA) metabolism, and protection against pathogens (8–10). The delivery of AA to the membrane-bound gp91^phox^ subunit boosts the activation of NADPH oxidase, generating reactive oxygen species (ROS) in phagocytes (11, 12). Extracellular S100A9 enhances transendothelial migration (13). S100A8/S100A9 also stimulates leukocyte migration by upregulating the expression of adhesion molecules and enhancing leukocyte–endothelial cell interaction (14–16). S100A8/S100A9 stimulate the production of TNFα and IL6 through ERK/NFκB and JNK/NFκB signaling (17). S100A9 can potentiate IL8 secretion induced by other neutrophil activators including fMLP and GMCSF, following NFκB, CREB-1, and STAT3/STAT5 activation (18). S100A8, S100A9, and S100A8/S100A9 have been demonstrated to modulate production of proinflammatory mediators, including cytokines, chemokines, ROS, and nitric oxide (19). Once released into the extracellular space from infiltrating phagocytes or after cell necrosis, the S100A8/S100A9 complex exhibits broad-spectrum antimicrobial activity against numerous microorganisms (20).

All these data support the important role of extracellular S100A8/S100A9 in regulation of the microenvironment and its potential involvement in tumor progression. Therefore, attempts were made to block S100A8/S100A9 in cancer. Tasquinimod (TQ), an oral quinoline-3-carboxamide, binds to S100A9 in the presence of Zn^2+^ and Ca^2+^ and blocks the interaction of S100A9 with its receptors, inhibiting TNFα release *in vivo* (21–23). TQ has recently emerged as a therapeutic agent in a limited number of experimental tumor models (22, 24–28). TQ prolonged the progression-free survival of patients with prostate cancer compared with placebo in both a phase II trial (29) and a randomized phase III trial (30), demonstrating that TQ is effective in delaying tumor progression. We hypothesized that S100A8/S100A9 may have a particularly important role in multiple myeloma growth given the abundance of MDSC in the myeloma BM TME and that TQ would therefore be effective at inhibiting multiple myeloma progression.

Here, we report that S100A8/S100A9 promotes multiple myeloma progression and that blockade of S100A9 with TQ has antimyeloma activity *in vivo*, as well as our surprising observation that part of the effect of S100A8/S100A9 on multiple myeloma progression is independent of the adaptive immune system and instead mediated by Toll-like receptor 4 (TLR4)- and STAT5-driven expansion of megakaryocytes (MK) and increased angiogenesis.

## Materials and Methods

### Human Samples

Collection of samples from patients with multiple myeloma at the Abramson Cancer Center at the University of Pennsylvania (Philadelphia, PA) was approved by the Institutional Review Board (IRB) of the University of Pennsylvania (Philadelphia, PA). Studies were conducted in accordance with the U.S. Common Rule. All patients signed IRB-approved consent forms.

### Cell Culture

The mouse myeloma cell lines DP42 and 5TGM1 (RRID:CVCL_VI66) were gifts from Dr. Van Ness (University of Minnesota, Minneapolis, MN) and Dr. Babatunde Oyajobi (The University of Texas Health Science Center at San Antonio, TX), respectively. The human multiple myeloma cell lines NCI-H929 (RRID:CVCL_1600), RPMI8226 (RRID:CVCL_0014), and MM1.S (RRID:CVCL_8792) were purchased from the ATCC and authenticated by short tandem repeat profiling. Upon receipt, cell lines were expanded, early passages were frozen, and vials were stored in liquid nitrogen. Cells used in all experiments were in culture for no longer than 2 months. All multiple myeloma cells were cultured in RPMI1640 medium (Corning) supplemented with l-glutamine, 10% FBS, and 50 units/mL of penicillin and 50 μg/mL of streptomycin. DP42 cells were also supplemented with 0.5 ng/mL murine IL6 (BioLegend). Cell lines were maintained at 37°C with 5% CO_2_ for a maximum of 6 weeks and were *Mycoplasma* negative as detected using Universal *Mycoplasma* Detection Kit (ATCC).

### Antibodies and Reagents

Antibodies used are listed in Supplementary Table S1. TQ was kindly provided by Active Biotech AB and formulated according to the manufacturer's instructions. S100A8, S100A9, and S100A8/S100A9 heterodimer proteins were kindly provided by Dr. Thomas Vogl (University of Münster, Münster, Germany) and Dr. Phillipe Tessier (Laval University, Quebec, Canada) and purchased from R&D Systems. The RAGE-antagonist FPS-ZM1 was purchased from Cayman Chemicals. Anti-CD147 blocking antibody was purchased from eBioscience (catalog no. 16-1471-82, RRID: AB_823121).

### MK Cultures

BM cells were isolated from mouse femurs and plated in DMEM containing Glutamax (Invitrogen) supplemented with 10% FBS (GE Hyclone), 50 units/mL of penicillin, and 50 μg/mL of streptomycin and indicated concentration of murine Thrombopoietin (TPO; BioLegend). Cells were incubated at 37°C with 5% CO_2_ for 5–6 days. Human MKs were cultured from CD34^+^ cells isolated from the BM of patients with multiple myeloma. Mononuclear cells were isolated using Ficoll-paque gradient centrifugation. CD34 cells were isolated using CD34 MicroBead kit (Miltenyi Biotech, RRID: AB_2848167) according to manufacturer's instructions. The isolated cells were plated in Iscove's modified Dulbecco's medium (Invitrogen) supplemented with 20% BIT9500 serum substitute and 1 × StemSpan Megakaryocyte Expansion Supplement (both from StemCell Technologies). The cultures were incubated at 37°C with 5% CO_2_ for 14 days. Cultured murine MKs were isolated using a BSA gradient as described previously (31).

### Collection of BM Cell Supernatants

Femur and tibia bones were collected from wild-type (WT) or S100A9 knockout (KO) mice and flushed with either 1 mL RPMI1640 media (used as supernatant for BM cells) or 500 μL PBS (used for soluble factors measurement) following by centrifugation at 1,500 rpm for 5 minutes. Cell pellets were discarded; supernatants were collected and used in experiments.

### Mouse Models and Treatment

All animal experiments were carried out in accordance with institutional guidelines and were approved by the Institutional Animal Care and Use Committee of The Wistar Institute (Philadelphia, PA). All mice were housed in pathogen-free conditions. Male and female mice ages 6–8 weeks were used in experiments. C57BL/6 and FVB/N mice were purchased from Charles River Laboratories and were crossed to obtain F1 progeny of mixed C57BL/6xFVB/N background. S100A9-deficient mice were described previously (32). Multiple myeloma tumors were established in these mice by intravenous inoculation of DP42 cells (5 × 10^3^ cells). RAGE KO mice were provided by Dr. John Hoidal (University of Utah, Salt Lake City, Utah). TLR4 KO (B6(Cg)-*Tlr4^tm1.2Karp^*/J, strain # 029015, RRID: IMSR_JAX:029015) mice were obtained from The Jackson Laboratory and C.B-17 scid mice from Charles River (CB17/lcr-*Prkdc^scid^*/lcrCr, RRID: IMSR_CRL:561). The colony of NSG (NOD.Cg-Prkdc*^scid^*Il2rg*^tm1Wjl^*/SzJ, RRID:IMSR_JAX:005557) mice was maintained in house. Tumors were established by subcutaneous injection of 10 × 10^6^ RPMI8226 or 5 × 10^6^ H929 cells into the right flanks of the mice or intravenous inoculation of 5 × 10^6^ RPMI8226 or MM1.S cells into the tail vein.

For *in vivo* treatment studies, DP42, H929, RPMI8226, or MM1.S-bearing mice were split into two groups and treated with TQ at a dose of 30 mg/kg *ad libitum* in drinking water. Treatment started on days 1–3 after tumor cell injection in DP42 and subcutaneous xenograft models, on day 5 in RPMI8226 intravenous model and on day 14 in MM1.S intravenous model. TQ was given continuously until mice reached the endpoint. Control mice received vehicle in drinking water. Survival of mice was determined as they were euthanized at the humane endpoint. In *in vivo* combination treatment studies with other agents, RPMI8226- and H929-bearing NSG mice were split into four groups and treated with TQ (30 mg/kg *ad libitum* in drinking water beginning 8 hours after tumor cell injection), lenalidomide (SelleckChem, 10 mg/kg, via gavage, 5 days on and 2 days off), bortezomib (Velcade, Takeda, 0.5 mg/kg, i.v., twice a week), ixazomib (SelleckChem, 5 mg/kg, via gavage, twice a week) or their combination. Treatment with anti-multiple myeloma agents started on days 13–14 after tumor cell injection. Growth of tumors on the flank was tracked with measurements using calipers twice a week. Mice were euthanized when tumors reached 400 mm^2^. Tumor growth rate was calculated for each mouse by determining the change in tumor size between initial measurement and measurement at the endpoint and dividing this difference by the number of days.

For *in vivo* overexpression experiments, mice were injected with 100 μg of each mouse *S100a8* and *S100a9*—encoding DNA plasmids pMV101-mS100A8-His and pMV101-mS100A9-His (synthesized by GenScript) in sterile water intramuscularly in the tibialis anterior muscle of both legs and subsequently electroporated using the CELLECTRA 3P adaptive electroporation device (Inovio Pharmaceuticals) as described previously (33). Mice were euthanized at indicated timepoints after *in vivo* electroporation and tissues were collected for analysis.

### Flow Cytometry

Single-cell suspensions were stained with Live/Dead Fixable Aqua Dead Cell Stain Kit (Invitrogen) and the indicated specific antibodies for 20 minutes at 4°C in the dark. For detection of apoptosis, Annexin V (BioLegend, catalog no. 640945)/DAPI staining was performed according to the manufacturer's protocol. At least 10,000 events were acquired using LSRII flow cytometer (BD Biosciences). Analyses were performed using FlowJo software (BD Biosciences). MK ploidy analysis was performed using propidium iodide staining.

### Quantitative Real-time PCR

RNA was extracted using E.Z.N.A. Total RNA purification kit (Omega Bio-Tek) and cDNA was synthesized using the High-Capacity cDNA Reverse Transcription Kit (Applied Biosystems). Quantitative PCR was performed using SYBR Green PCR Master Mix and primers listed in Supplementary Table S2 using a QuantStudio6 Flex real-time PCR instrument (Applied Biosystems). Expression of specific genes was normalized to the expression of a housekeeping gene β-actin.

### Western Blotting

Cells were lysed using RIPA buffer (Sigma-Aldrich) supplemented with 1 × Halt Protease and Phosphatase Inhibitor cocktail (Thermo Fisher Scientific). Lysates were subjected to 10% SDS-PAGE followed by transfer to nitrocellulose membrane (Bio-Rad). Membranes were blocked with 5% nonfat dry milk and incubated with primary antibodies overnight at +4°C followed by incubation with secondary horseradish peroxidase–conjugated antibodies for 2 hours at room temperature. The following primary antibodies were used: anti-His-tag (RRID: AB_2744546), anti-S100A9 (RRID: AB_2734726), anti-phospho-STAT5 (Tyr694) (RRID: AB_10544692), anti-STAT5 (RRID: AB_2737403; Cell Signaling Technology), anti-β-actin (Santa Cruz Biotechnology, RRID: AB_626632).

### ELISAs

Levels of S100A8/S100A9 proteins and TPO were measured using human S100A8/S100A9 and mouse S100A8/S100A9 heterodimer ELISA kits and mouse TPO ELISA kit (R&D Systems) according to manufacturer's instructions.

### IHC

Mouse femurs were isolated and fixed overnight in 10% formalin. The bones were then placed in a 70% ethanol solution for at least overnight before being decalcified and embedded in paraffin. Sections were cut and stained with hematoxylin and eosin (H&E) or anti-CD31 antibody (Santa Cruz Biotechnology, RRID: AB_2161037).

### Statistical Analysis

Statistical analysis was performed using GraphPad Prism 9 software. Tumor growth rate for each animal was calculated by its tumor size at last follow-up minus tumor size at starting treatment with antimyeloma agents and then divided by the days during this period. The average tumor growth rates were compared between groups. Differences between two experimental groups were determined using two-tailed Student *t* test or Mann–Whitney test. To compare three or more groups, ANOVA with *post hoc* Tukey for between group comparisons were performed. Differences in kinetics of tumor growth were analyzed using two-way ANOVA. Survival was analyzed using log-rank test. *P* value of less than 0.05 was considered statistically significant.

### Data Availability

The data generated in this study are available within the article and its Supplementary Data.

## Results

### Multiple Myeloma Progression is Associated with Increased Production of S100A8/S100A9 Proteins

**FIGURE 1:**
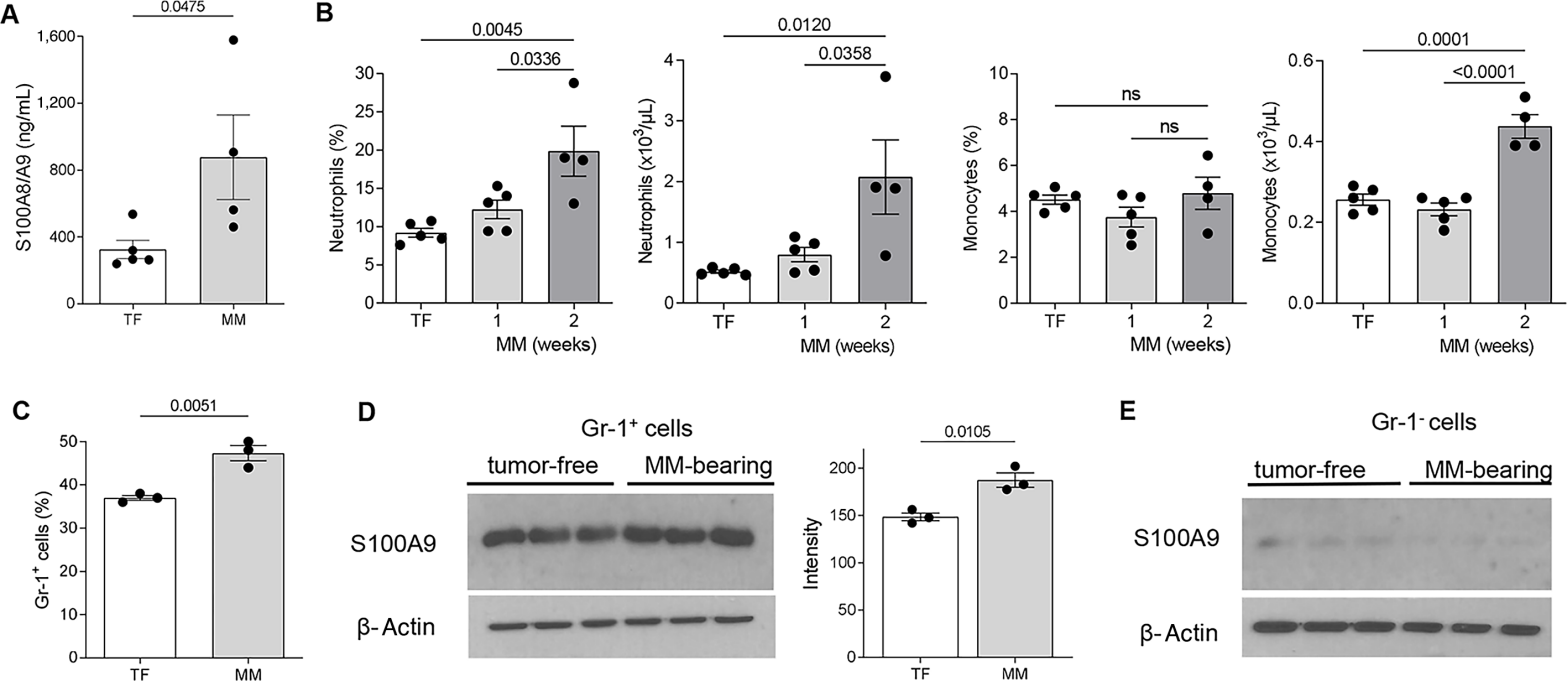
S100A8/A9 level is increased in multiple myeloma (MM)-bearing mice. **A,** Levels of S100A8/A9 heterodimer in BM supernatants from tumor-free (TF) and DP42-bearing mice were measured by ELISA. **B,** Frequency and absolute number of neutrophils and monocytes in peripheral blood of TF and DP42-bearing mice 1 and 2 weeks after tumor cell inoculation were determined by a complete blood count. **C,** Frequencies of CD11b^+^Gr-1^+^ cells were evaluated in the BM of TF and DP42-bearing mice using flow cytometry. Levels of S100A9 protein were determined by Western blot analysis in Gr-1^+^ BM cells (**D**) and Gr-1^−^ BM cells (**E**) isolated from TF and DP42-bearing mice. Loading was confirmed by reprobing the membranes with antibody against β-actin. Representative Western blot results (**D**, left and **E**) and quantitation of S100A9 expression normalized to the expression of β-actin (**D**, right) are shown. Individual values, mean, and SEM values are shown. Statistics: two-tailed Student *t* test (**A**, **C**, **D**) and ANOVA with Tukey multiple comparisons test (**B** and **C**).

To investigate the involvement of S100A8/S100A9 in multiple myeloma progression, we evaluated the amount of these proteins in the BM of tumor-free and DP42 myeloma-bearing mice. A syngeneic DP42 model of myeloma was established by intravenous injection of tumor cells that home and grow in the BM (1). Two weeks after tumor inoculation, BM from DP42-bearing mice had a significantly higher amount of S100A8/S100A9 than tumor-free mice (Fig. 1A). Because myeloid cells are the main sources of S100A8/S100A9 (34–36), we asked whether tumor growth was associated with an increase in the number of these cells. Consistent with previous observations (37), blood cell count demonstrated a significant increase in the presence of neutrophils and monocytes in the peripheral blood of mice 2 weeks after inoculation of DP42 tumor (Fig. 1B), as well as an increase in the proportion of CD11b^+^Gr-1^+^ MDSC in the BM (Fig. 1C). In addition to the increased number, CD11b^+^Gr-1^+^ cells from the BM of multiple myeloma–bearing mice produced higher amounts of S100A9 protein (Fig. 1D), which is consistent with the accumulation of MDSC in tumor-bearing mice because MDSC are known to express more S100A9 protein than neutrophils and monocytes (36). No differences were found in the production of S100A9 by Gr-1^−^ BM cells between tumor-free and multiple myelom–bearing mice (Fig. 1E). Thus, the higher level of S100A8/S100A9 in the BM of multiple myeloma–bearing mice was associated the accumulation of MDSC in the presence of tumor cells and increased production of S100A8/S100A9 by these MDSC.

### S100A9 Induces Expansion of MKs

**FIGURE 2:**
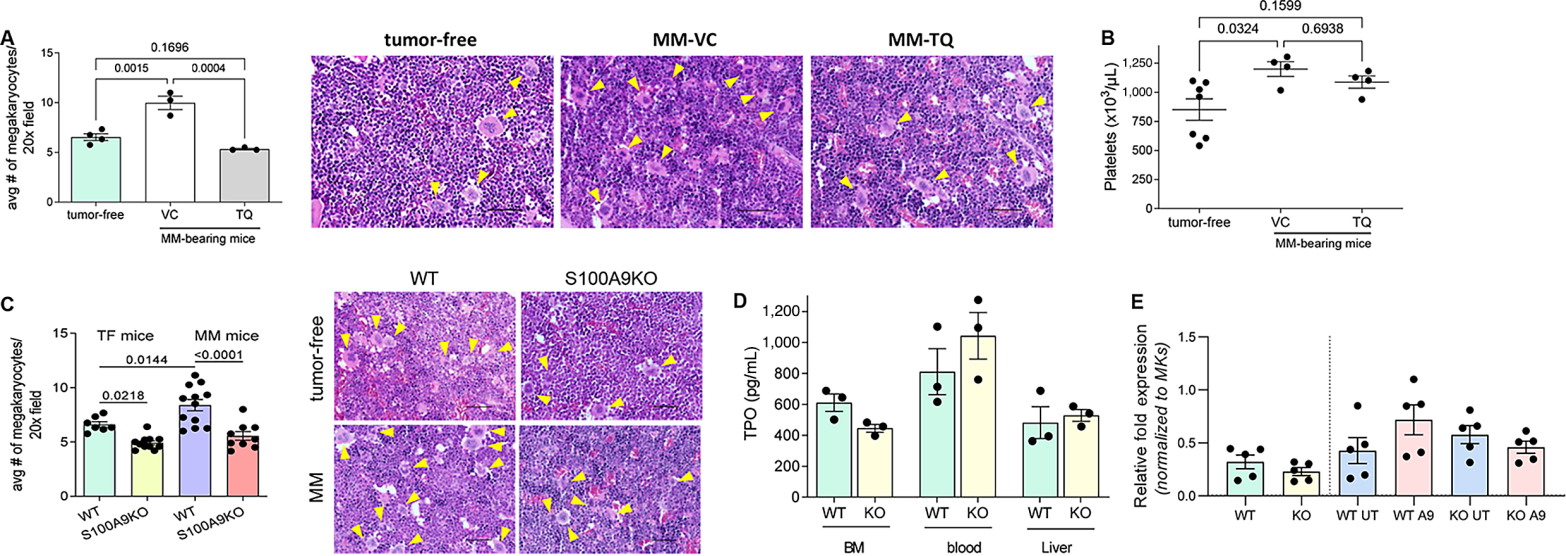
S100A9 protein induces expansion of MKs. **A–C,** DP42 tumors were established in syngeneic mice by intravenous injection of tumor cells. Treatment with TQ started on day 3 after tumor cell injection. **A,** Number of MKs was counted in the BM from tumor-free and DP42-bearing mice using H&E-stained slides. **B,** Number of platelets measured by complete blood count is shown. **C,** Number of MKs in the BM of WT and S100A9KO tumor-free (TF) and DP42-bearing mice was counted using H&E-stained slides. Cumulative results (left) and representative images (right) are shown (**A** and **C**). Magnification, 20x. Scale bar, 50 μm. Yellow arrows indicate MKs. **D,** Levels of TPO were measured by ELISA in the BM supernatants, blood, or liver lysates collected from WT and S100A9KO mice. **E,** Expression of c-Mpl was determined by qPCR in BM cells either freshly isolated from WT and S100A9KO mice (WT and KO) or cultured for 2 days with (A9) or without (UT) addition of S100A9 protein. Individual values, mean, and SEM values are shown. Statistics: one-way ANOVA with Tukey multiple comparisons test (**A**–**C**), two-tailed Student *t* test (**D** and **E**).

Multiple myeloma progression was associated with a significant increase in the presence of MKs in the BM, which was abrogated by TQ (Fig. 2A). An increase in MKs was associated with elevated level of platelets in blood of multiple myeloma–bearing mice, an effect not observed in TQ-treated mice (Fig. 2B). Interestingly, no effect of TQ on megakaryopoiesis was found in tumor-free mice (Supplementary Fig. S1). To better clarify the role of S100A8/S100A9 in the expansion of MKs, we used S100A9 KO mice. Tumor-free and DP42 multiple myeloma–bearing S100A9 KO mice displayed significantly lower numbers of MKs than their WT counterparts (Fig. 2C). TPO is the main growth factor responsible for MK expansion and differentiation (38). We tested the possibility that S100A8/S100A9 affects the expression of TPO or its main receptor c-Mpl and thus causes an increase in MKs. However, no decrease in TPO (Fig. 2D) or c-Mpl (Fig. 2E) was observed in S100A9 KO mice.

**FIGURE 3:**
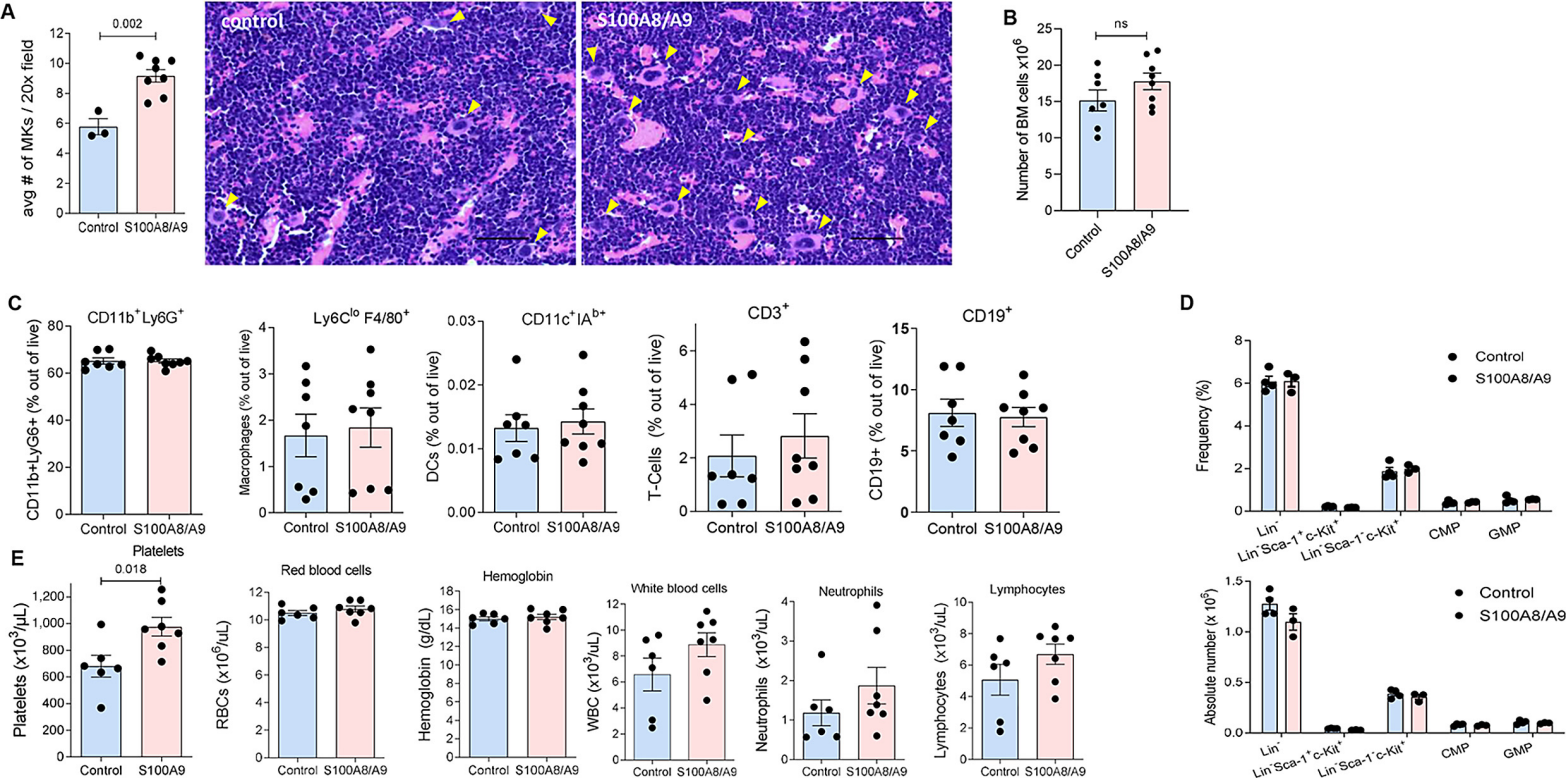
*In vivo* expression of S100A8/S100A9 induces megakaryopoiesis. S100A8/S100A9 proteins were expressed in S100A9KO mice by *in vivo* electroporation of *S100a8* and *S100a9* plasmids. In control, mice were electroporated with empty vector. Mice were euthanized 14 days after the procedure. **A,** Number of MKs in the BM was counted using H&E-stained slides. Representative images and cumulative results are shown. Arrows indicate MKs. Magnification, 20x. Scale bar, 50 μm. **B,** Number of BM cells was determined by counting cells from two femurs per each mouse. **C,** Proportion of indicated BM cell populations was determined by flow cytometry. **D,** Proportion and absolute number of indicated BM cell populations are shown. **E,** Results of complete blood count are shown. Presented are individual values, mean, and SEM values. Statistics: two-tailed Student *t* test.

To further verify the role of S100A8/S100A9 proteins in MK expansion, tumor-free S100A9 KO mice were electroporated intramuscularly with plasmids expressing mouse *S100a8 and S100a9*. This resulted in the expression of these proteins in the BM (Supplementary Fig. S2), which lasted for at least 12 days. Induced expression of S100A8 and S100A9 in S100A9 KO mice caused a significant increase in the number of MKs in the BM (Fig. 3A). However, no changes in the total number of BM cells were observed (Fig. 3B) and no differences in the proportion of various myeloid and lymphoid cells (Fig. 3C) and myeloid progenitors (Fig. 3D) were evident in the BM. We also evaluated the peripheral blood of these mice and found that expression of S100A8 and S100A9 increased the number of platelets but not red cells, white blood cells, neutrophils, or lymphocytes (Fig. 3E). These results indicate that S100A8 and S100A9 have a selective effect on the differentiation of MKs.

**FIGURE 4:**
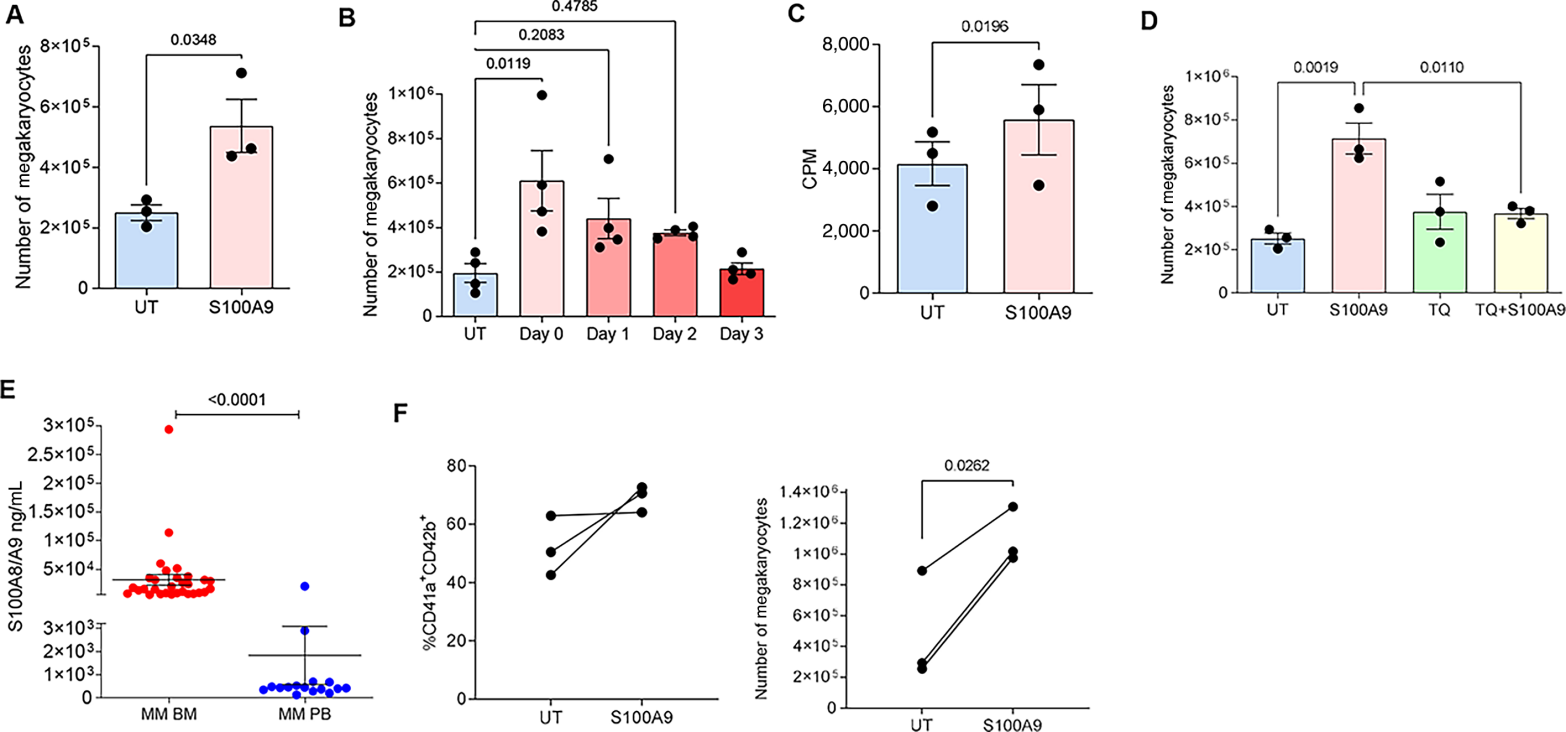
S100A9 induce expansion of MKs. **A** and **B,** Effect of S100A9 protein on mouse MK differentiation. BM cells were cultured in the presence of TPO and S100A9 protein added on day 0 (**A**) or indicated days (**B**) of culture. Cells were collected on day 5 and number of CD41^+^ MKs was determined. **C,** HPC were cultured with or without S100A9 for 48 hours followed by measuring incorporation of 3[H]Thymidine. **D,** BM cells cultured with TPO in the presence or absence of S100A9 protein were treated with 10 μmol/L TQ added on day 0. Number of MKs was determined on day 5 of cultures. **E,** Level of S100A8/A9 protein in BM (*n* = 32) and PB (peripheral blood; *n* = 16) plasma of patients with multiple myeloma. **F,** CD34^+^ cells isolated from BM of patients with multiple myeloma were differentiated toward MKs in the presence or absence of hS100A9 protein. Proportion and number of CD41a^+^CD42b^+^ MKs was determined by flow cytometry on day 12 of cultures. UT, untreated (no S100A9 protein added). Individual values, mean, and SEM values are shown. Statistics: two-tailed Student *t* test (**A, C,** and **F**), one-way ANOVA with Tukey multiple comparison test (**B** and **D**), and Mann–Whitney test (**E**).

To better understand the role of S100A9 protein in MK differentiation, BM progenitors were differentiated for 5 days in the presence of TPO and recombinant S100A9. In preliminary experiments, we determined the optimal concentration of TPO required for MK differentiation. The number of MK reached a plateau when TPO was used at a concentration of 1 ng/mL (Supplementary Fig. S3A). The addition of recombinant S100A9 increased differentiation of CD41^+^ MKs from BM progenitors (Fig. 4A). Without TPO, S100A9 was insufficient to support MK differentiation (Supplementary Fig. S3B). The effect of S100A9 on MK differentiation was observed only when it was added in the first 1–2 days of the culture, but not later during the culture (Fig. 4B). Addition of S100A9 to BM progenitors differentiated in the presence of TPO caused a modest but significant increase in cell proliferation during the first 48 hours in culture (Fig. 4C). TQ abrogated the effect of S100A9 on MK differentiation (Fig. 4D). While the number of MK was increased, no changes in MK ploidy were observed in the presence of S100A9 protein or the S100A8/S100A9 heterodimer (Supplementary Fig. S4A).

MKs are known to produce cytokines that support angiogenesis (39, 40). We tested the effect of S100A9 on the expression of genes encoding cytokines produced by MKs. MKs differentiated *in vitro* in the presence of S100A9 had markedly higher expression of *Vegf*, but not *Fgf2, Tsp1, Il6, or Il1b.* No differences in the expression of TPO receptor *c-Mpl* were observed (Supplementary Fig. S4B). The effect of S100A9 was specific to MKs, as S100A9 did not affect the expression of cytokines by stroma cells generated from the BM of tumor-free mice (Supplementary Fig. S4C) or upregulate expression of *Vegf* in multiple myeloma cells (Supplementary Fig. S4D).

We assessed the role of S100A8/S100A9 proteins in BM angiogenesis in the model of multiple myeloma. It is known that multiple myeloma is associated with increased angiogenesis (41). We confirmed this phenomenon by observing an increase in CD31^+^ endothelial cells in the BM of DP42-bearing mice. This increase was markedly diminished by the treatment with TQ (Supplementary Fig. S5).

Next, we evaluated the presence of S100A8/S100A9 protein in patients with multiple myeloma. The amount of S100A8/S100A9 in BM was markedly higher than in peripheral blood (Fig. 4E). Human CD34^+^ hematopoietic progenitor cells were isolated from the BM of patients with multiple myeloma and differentiated to MKs with or without S100A9. Out of CD34^+^ cells that grow, and similar to the effect observed in mice, the addition of S100A9 significantly increased differentiation of MKs (Fig. 4F). Thus, these results indicate that S100A9 can drive expansion of MKs in both mice and humans.

### Mechanism of S100A9 Effect on MK Differentiation

**FIGURE 5:**
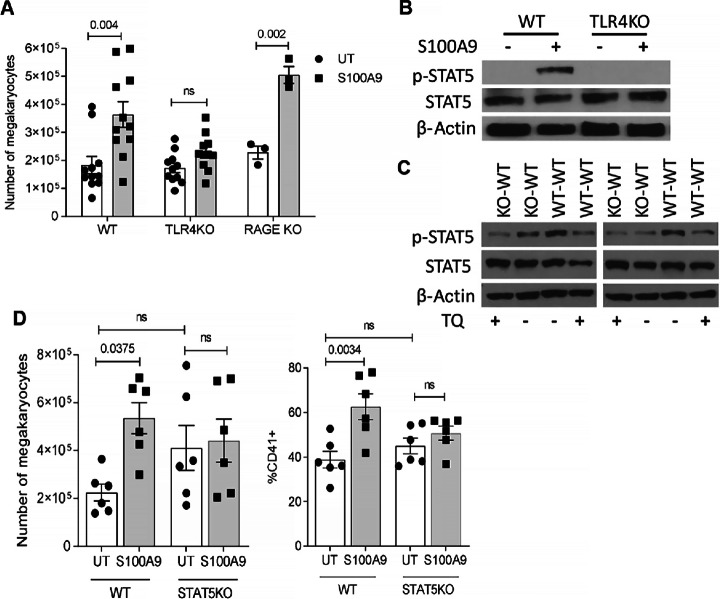
Mechanism of S100A9 effect on MK differentiation. **A,** BM cells were isolated from WT, TLR4 KO, or RAGE KO mice and cultured in the presence of TPO with or without S100A9. Number of MKs was determined on day 5 of culture. **B,** BM cells from WT or TLR4 KO mice were kept in serum-free media with or without S100A9 for 2 hours. Activation of STAT5 was evaluated by Western blotting. **C,** WT BM cells were cultured in the presence of supernatants collected from BM cells from WT mice (WT-WT) or BM cells from S100A9 KO mice (KO-WT) without addition of TPO with or without 10 μmol/L TQ. Activation of STAT5 was evaluated by Western blotting. Experiment was repeated three times with similar results; results of two of them are shown. **D,** BM cells from WT or STAT5 KO mice were cultured in the presence of TPO with or without S100A9 protein for 5 days. Absolute number and proportion of MK were determined. Individual values, mean, and SEM values are shown. Statistics: ANOVA with Tukey multiple comparisons test.

Three main receptors have been previously implicated in various biological effects of S100A8/S100A9: TLR4, RAGE, and CD147 (34). To determine the possible role of these receptors in S100A9-mediated MK expansion, BM cells from WT, TLR4-, or RAGE-deficient mice were cultured with S100A9 and TPO for 5 days. S100A9 caused a significant accumulation of MKs differentiated from WT BM cells. This effect was absent in BM cells from TLR4 KO mice (Fig. 5A). In contrast, in BM progenitors from RAGE KO mice S100A9 promoted MK differentiation, similarly to WT cells (Fig. 5A). To verify this observation, we used the RAGE inhibitor FPS-ZM1. At concentrations known to block RAGE signaling (42), the inhibitor did not prevent MK differentiation induced by S100A9 (Supplementary Fig. S6A). To block CD147 interaction with S100A9, we used a neutralizing antibody. Although S100A9 caused a modest increase in the number of MKs generated in the presence of CD147 antibody, it was not statistically significant from the number of MK generated in the absence of S100A9 (Supplementary Fig. S6B). However, these results need to be interpreted cautiously, considering that in the absence of S100A9 protein, the anti-CD147 antibody itself caused a substantial increase in MKs compared with isotype, probably through a separate, yet-to-be-identified mechanism. These data make it difficult to conclude that CD147 mediates the effect of S100A9 on MKs. Thus, it appears that the effect of S100A9 on MK expansion is regulated largely via TLR4.

MK differentiation is mainly controlled by the STAT5 transcription factor (42). Phosphorylation of STAT5 at Tyr694 (pSTAT5) is a critical step in its activation. BM cells were isolated from WT and TLR4 KO mice and incubated with S100A9 for 2 hours in the absence of TPO. In WT cells, S100A9 caused a marked increase in pSTAT5, whereas this effect was absent in TLR4-deficient cells (Fig. 5B). These results indicate that S100A9 can directly upregulate activation of STAT5 via TLR4. Because S100A9 is released primarily by neutrophils and monocytes, we asked whether supernatant from S100A9 KO BM cells could recapitulate this effect. We collected supernatants from BM cells isolated from WT and S100A9 KO mice. Treatment of WT BM cells with WT BM cell supernatant in the absence of TPO and without TQ caused activation of STAT5 (WT-WT; Fig. 5C). The presence of TQ substantially reduced this effect. Similarly, activation of STAT5 was reduced in BM cells cultured with supernatant from S100A9 KO mice and this effect was unchanged by addition of TQ (Fig. 5C). These results support the role of S100A9 in the upregulation of STAT5 activation in BM cells. We then asked whether STAT5 signaling downstream of S100A9 mediated its effect on MK differentiation. BM progenitors from STAT5^fl/fl^ RosaCre^+^ and control STAT5^fl/fl^RosaCre^−^ littermates were cultured with recombinant S100A9 protein for 5 days in the presence of TPO. Consistent with previous results, S100A9 caused a significant increase in MK differentiation from BM cells isolated from control STAT5^fl/fl^RosaCre^−^ mice. In contrast, no increase in megakaryopoiesis was observed during differentiation of STAT5-deficient progenitors (Fig. 5D). These results support the hypothesis that S100A9 induces MK differentiation via upregulation of STAT5.

### TQ has a Potent Immune System-independent Antitumor Effect in Models of Multiple Myeloma

**FIGURE 6:**
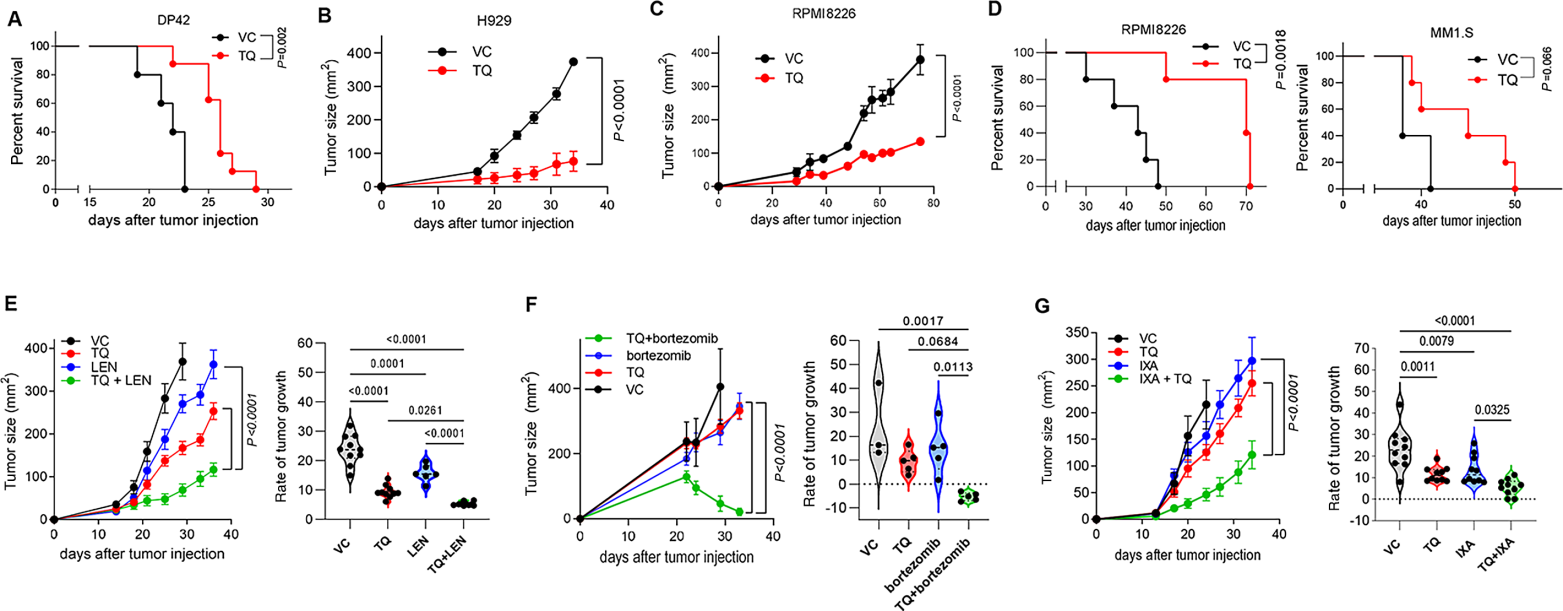
TQ demonstrates potent antitumor effect in multiple myeloma *in vivo* models. **A,** Survival of DP42-bearing mice treated with TQ (30 mg/kg/day, *n* = 8) or vehicle control (VC, *n* = 5). **B,** Growth kinetics of human H929 subcutaneous tumors established in C.B-17 SCID mice and treated with TQ (*n* = 4) or VC (*n* = 4). **C,** Growth kinetics of human RPMI8226 subcutaneous tumors established in NSG mice and treated with TQ (*n* = 5) or VC (*n* = 4). **D,** Human MM RPMI8226 or MM1.S tumors were established in NSG mice by intravenous injection of 5 × 10^6^ tumor cells. Treatment with TQ or VC started on day 14 after tumor cell injection. Survival of mice was determined. *n* = 5 per group. Statistics: two-way ANOVA (**B** and **C**) or log-rank test (**A** and **D**). **E–G**, Human multiple myeloma H929 tumors were established in NSG mice by subcutaneous injection of 5 × 10^6^ cells. Mice were split into groups and given either TQ, lenalidomide (LEN, **E**), bortezomib (**F**), or ixazomib (IXA, **G**) as monotherapy or in combination with TQ. Kinetics of tumor growth (left) and tumor growth rate (right) are shown. Statistics: ANOVA with Tukey multiple comparisons test.

To determine a possible biological role of S100A8/S100A9 in multiple myeloma, we assessed the therapeutic effect of TQ in different models of multiple myeloma. A syngeneic DP42 model of multiple myeloma was established by intravenous injection of tumor cells that home and grow in the BM (1). Treatment with TQ significantly increased the survival of mice (Fig. 6A). At least some of the antimyeloma effect of TQ was independent of the adaptive immune system, as a significant antitumor effect of TQ was also observed in xenograft models of human multiple myeloma, including a subcutaneous model of H929 grown in C.B-17 scid mice lacking T cells (Fig. 6B), a subcutaneous model of RPMI8226 multiple myeloma (Fig. 6C), and intravenous models of RPMI8226 and MM1.S MM (Fig. 6D) grown in NSG mice lacking T, B, and natural killer cells. In these immunodeficient models, TQ markedly potentiated the antitumor effects of several therapeutics commonly used in multiple myeloma, including lenalidomide and the proteasome inhibitors bortezomib and ixazomib (Fig. 6E–G). To demonstrate that the effect of TQ is due to blockade of S100A9, we tested TQ in S100A9 KO mice, which lack both S100A8 and S100A9 proteins (43). No antitumor effect of TQ was detected in the absence of these proteins (Supplementary Fig. S7). To determine whether TQ has a direct effect on myeloma cells, we tested TQ in multiple myeloma cell lines grown *in vitro* in the absence of a supportive BM microenvironment; in all tested cell lines, TQ had no effect on tumor cell growth (Supplementary Fig. S8A) or cell death (Supplementary Fig. S8B). Cells of BM TME did not induce S100A9 production by multiple myeloma cells (Supplementary Fig. S8C). Taken together, these data demonstrate that blocking S100A9 has potent antitumor effect. This effect appears to be mediated through modulation of the BM microenvironment and at least partly is independent of the adaptive immune system.

## Discussion

Our study demonstrated a significant role of the myeloid-derived proteins S100A8/S100A9 in the progression of multiple myeloma. Blockade of S100A9 with TQ had a potent antitumor effect and markedly potentiated the effects of several commonly used anti-multiple myeloma therapeutics in several multiple myeloma mouse models.

We demonstrated that S100A8/S100A9 directly promote differentiation of MKs and implicated TLR4 and STAT5 in this process. The effect of S100A8/S100A9 on MKs was observed in “gain of function” and “loss of function” settings *in vivo* and *in vitro*.

We believe that S100A8/S100A9 may have a broad effect on hematopoietic differentiation across different cell populations, but its effect may be more detectable in the number of MKs due to their relative infrequency in the BM. We found that S100A8/S100A9 did not drive differentiation or accumulation of neutrophils, a major population of myeloid cells, or lymphocytes. However, it is likely this selectivity was not due to a unique biochemical process associated with S100A8/S100A9 signaling, but rather due to the fact that the baseline number of neutrophils and other white and red blood cells is much higher than the number of MKs, and thus changes in these cells are much more difficult to detect. As a result, the increased amount of S100A8/S100A9 in multiple myeloma BM was not sufficient to cause a detectable effect on myeloid cells. Thus, regulation of MKs can be a unique feature of the effect of S100A8/S100A9 proteins in BM. MKs were not only expanded with S100A8/S100A9 but were also activated to express more *Vegf*, which is critical to support angiogenesis. An increased number of platelets in peripheral blood also depended on the presence of S100A8/S100A9, supporting the involvement of MKs in the observed phenomenon.

MK progenitors can be generated not only from MK-erythroid progenitors (MEP), but also directly from multipotent progenitor (primarily MPP2), or long-term hematopoietic stem cell (38). It appears that S100A8/S100A9 affected late precursors of MK because no changes in CMP or MEP were observed in mice overexpressing S100A8/S100A9.

An increased number of BM CD11b^+^Gr1^+^ cells (MDSC) as well as increased production of S100A8/S100A9 by these cells contributed to the elevated levels of S100A8/S100A9 proteins in multiple myeloma–bearing hosts. These data are in disagreement with previously reported results showing no difference in production of S100A9 protein between neutrophils and monocytes from tumor-free and 5T33 multiple myeloma–bearing mice (44). The lack of such differences in previously reported results could be explained by insufficient purity of isolated populations of myeloid cells or distinct features of the 5T33 multiple myeloma model (perhaps also explaining the lack of significant antitumor effect of blocking S100A9 with ABR-238901 in that study). However, the antitumor effect was evaluated by measuring the presence of idiotype-positive cells, and no information on total disease burden or mice survival after S100A9-blocking treatment was provided (44). These data would be critically important to understand the potential antitumor effect of blocking S100A9 in the 5T33 multiple myeloma model.

The levels of S100A8/S100A9 in the BM of patient with multiple myeloma were significantly elevated when compared with those in peripheral blood of these patients as well as to the S100A8/S100A9 levels reported in healthy donors (45, 46). Previous studies demonstrated that MKs are expanded in BM areas infiltrated with multiple myeloma and promoted proliferation of multiple myeloma cells (47, 48). However, mechanisms governing this expansion are not well understood. Our work provided first evidence that S100A8/S100A9 proteins are at least partially responsible for the supporting megakaryopoiesis in the myeloma BM. However, more studies are needed to better understand a clinical significance of S100A8/S100A9 within the established multiple myeloma—myeloid–MK axis that may regulate megakaryopoiesis in patients with multiple myeloma.

Interestingly, we did not observe the blocking effect of TQ on megakaryopoiesis in tumor-free mice. Recent data demonstrated that while S100A8/S100A9 dimers bind and mediate their effects via TLR4, formation of S100A8/S100A9 tetramers induces conformational changes that prevent binding to this receptor (49). There is a possibility that in tumor-free mice, S100A8/S100A9 exist mainly in the form of tetramers and that TQ is unable to bind S100A9 and block its effects.

Intracellular S100A8/S100A9 is involved in the suppressive activity of MDSC and macrophages (50, 51). In contrast, a direct inhibitory effect of S100A8/S100A9 on T cells is not widely observed. S100A8/S100A9 can regulate immune suppression in the TME indirectly via regulation of MDSC (52). Our demonstration that the antitumor effect of TQ was observed in immune deficient mice supports the notion that S100A8/S100A9 proteins exert their effects not only through regulation of adaptive immunity, but through other mechanisms. In addition to blocking S100A9, TQ has been previously reported to bind HDAC4 and inhibit its activity (53); this could be an additional mechanism by which treatment with TQ delayed multiple myeloma progression. However, our data showing lack of antitumor effect of TQ in S100A9 KO mice suggest that blocking S100A9 interaction with its receptors may represent the main mechanism by which TQ mediates its effect *in vivo.* Nevertheless, evaluation of HDAC4 function in multiple myeloma–bearing WT and S100A9 KO mice during disease progression as well as *in vitro* studies demonstrating dose-dependent response to TQ would contribute to better understanding the role of S100A8/S100A9 proteins in multiple myeloma biology. Ghavami and colleagues demonstrated that S100A8/S100A9 proteins used at a concentration of 10 μg/mL induced growth of several breast tumor cell lines including MCF-7, MDA-MB231, and SHEP, whereas at higher concentrations it did not enhance cellular proliferation (54). Low concentrations of S100A9 promote cell proliferation/survival of both normal intestinal epithelial cells and tumor cells (55). We did not observe similar effects in multiple myeloma cell lines. Further studies aimed to identify the predominant mechanism of TQ especially in clinical setting are warranted.

The BM microenvironment in multiple myeloma is characterized by increased microvessel density. The production of proangiogenic molecules is increased and the production of angiogenic inhibitors is suppressed, leading to an “angiogenic switch.” Current data suggest that the increased BM angiogenesis in multiple myeloma is due to the aberrant expression of angiogenic factors by multiple myeloma cells and cells in the TME (56). A previous study implicated angiogenesis as a possible mechanism of S100A9 effect (44). S100A9 induced MDSC to express and secrete inflammatory and pro-multiple myeloma cytokines, including TNFα, IL6, and IL10. Blocking S100A9 interactions *in vivo* with the small-molecule inhibitor ABR-238901 reduced angiogenesis (44). The mechanisms of the proangiogenic effect of S100A9 remain unclear. Interestingly, in another study, S100A9 potentiated IL8 secretion induced by other neutrophil activators including fMLP and GMCSF, following NFκB, CREB-1, and STAT3/STAT5 activation (18).

Our results demonstrated a novel mechanism by which S100A8/S100A9 can regulate BM TME in multiple myeloma via upregulation of MKs. MKs are not only precursors of platelets but are known to regulate production of multiple cytokines that promote angiogenesis. Our data suggest that targeting S100A9 with TQ has strong antitumor effects that are mediated by a blockade of MK expansion and inhibition of angiogenesis. This effect, which is independent of the adaptive immune system, supports the further development of S100A9 inhibition with TQ in the clinic.

## Supplementary Material

Supplementary Tables S1 and S2 Supplementary Table 1. Antibodies used in the study.
Supplementary Table 2. Primers used in the study.

Figure S1 MK count in the BM of tumor-free mice treated with TQ.

Figure S2 Expression of S100A8 and S100A9 proteins using in vivo electroporation technique.

Figure S3 Effect of TPO and S100A9 on in vitro MK differentiation.

Figure S4 Effect of S100A9 protein on mouse MKs.

Figure S5 BM angiogenesis in tumor-free and MM-bearing mice treated with TQ.

Figure S6 Mechanism of S100A9 effect on MK differentiation.

Figure S7 Effect of TQ in S100A9KO MM-bearing mice.

Figure S8 Effect of TQ on MM cells.
