# Supplementary material for: S100A8/S100A9 Promote Progression of Multiple Myeloma via Expansion of Megakaryocytes
Source: Cancer Res Commun. 2023 Mar 13;3(3):420–30. doi: 10.1158/2767-9764.CRC-22-0368 (PMC10010194; doi:10.1158/2767-9764.CRC-22-0368)
Supplement: Supplementary Tables S1 and S2 — Supplementary Table 1. Antibodies used in the study. Supplementary Table 2. Primers used in the study. [file crc-22-0368-s01.docx]

**Supplementary Table 1. Antibodies used in the study.**

| **Name** | **Manufacturer** | **Cat. number** | **RRID** |
| --- | --- | --- | --- |
| APC/Cy7 anti-mouse/human CD11b | BioLegend | 101226 | AB_830642 |
| APC/Cy7 rat anti-mouse Gr1 | BioLegend | 108423 | AB_2137486 |
| FITC anti-mouse CD41 | BioLegend | 133904 | AB_2129746 |
| PE anti-mouse CD41 | BioLegend | 133906 | AB_2129745 |
| PerCP/Cy5.5 anti-mouse Ly6C | BioLegend | 128012 | AB_1659241 |
| PE/Cy7 anti-mouse Ly6G | BioLegend | 127618 | AB_1877261 |
| PE anti-mouse F4/80 | BioLegend | 123110 | AB_893486 |
| APC rat anti-mouse CD11c | BioLegend | 117310 | AB_313779 |
| PE/Cy7 anti-mouse I-A/I-E | BioLegend | 107630 | AB_2069376 |
| PerCP/Cyanine5.5 rat anti-mouse CD3 | BioLegend | 100218 | AB_1595492 |
| BV 421 rat anti-mouse CD4 | BioLegend | 100438 | AB_11203718 |
| APC/Cyanine7 rat anti-mouse CD8 | BioLegend | 100713 | AB_312752 |
| PerCP/Cy5.5 anti-mouse/human CD45R/B220 | BioLegend | 103236 | AB_893354 |
| APC/Cy7 anti-mouse CD19 | BioLegend | 152411 | AB_2922473 |
| Biotin anti-mouse Lineage Panel | BioLegend | 133307 | AB_11124348 |
| PE/Cy7 anti-mouse Ly6A/E (Sca-1) | BioLegend | 108114 | AB_493596 |
| PE anti-mouse CD117 (c-kit) | BioLegend | 105808 | AB_313217 |
| APC rat anti-mouse CD34 | BioLegend | 119310 | AB_1236469 |
| APC/Cyanine7 rat anti-mouse CD16/32 | BioLegend | 101328 | AB_2104158 |
| FITC mouse anti-human CD41a | BioLegend | 303704 | AB_314374 |
| APC mouse anti-human CD42b | BioLegend | 303912 | AB_2113770 |
| CD147 monoclonal antibody, functional grade | eBioscience | 16-1471-82 | AB_823121 |
| Purified rat IgG2a kappa isotype control | BioLegend | 400502 | AB_326523 |
| PECAM-1 (CD31) | Santa Cruz | sc-1506 | AB_2161037 |
| Phospho-STAT5 (Tyr694) | Cell Signaling | 4322 | AB_10544692 |
| STAT5 (D206Y) | Cell Signaling | 94205 | AB_2737403 |
| His-Tag | Cell Signaling | 12698 | AB_2744546 |
| S100A9 | Cell Signaling | 72590 | AB_2734726 |
| β-actin | Santa Cruz Biotechnology | sc-47778 | AB_626632 |

**Supplementary Table 2. Primers used in the study.**

| Gene | 5’ – 3’ | 3’ – 5’ |
| --- | --- | --- |
| *Gapdh* | ATGGTGAAGGTCGGTGTGAA | AATGAAGGGGTCGTTGATGG |
| *c-Mpl* | CATCCTTGTAGAGGTGACCACAG | TCCAGCCTTCCACTTGAGAC |
| *Tpo* | GGCCATGCTTCTTGCAGTG | AGTCGGCTGTGAAGGAGGT |
| *Cf3* | AACCCACCAACTATACCTACACT | GTCTGTGAGGTCGCACTCG |
| *Vegf* | CAGGCTGCTGTAACGATGAA | AATGCTTTCTCCGCTCTGAA |
| *Fgf2* | gcgacccacacgtcaaacta | tcccttgatagacacaactcctc |
| *Tsp1* | ggggagataacggtgtctttg | Cggggatcaggttggcatt |
| *Il6* | tagtccttcctaccccaatttcc | Ttggtccttagccactccttc |
| *Il1b* | AACCTGCTGGTGTGTGAQCGTTC | CAGCACGAGGCTTTTTTGTTGT |
