# Supplementary material for: S100A8/S100A9 Promote Progression of Multiple Myeloma via Expansion of Megakaryocytes
Source: Cancer Res Commun. 2023 Mar 13;3(3):420–30. doi: 10.1158/2767-9764.CRC-22-0368 (PMC10010194; doi:10.1158/2767-9764.CRC-22-0368)
Supplement: Figure S1 — MK count in the BM of tumor-free mice treated with TQ. [file crc-22-0368-s02.pdf]

Supplementary Figure S1

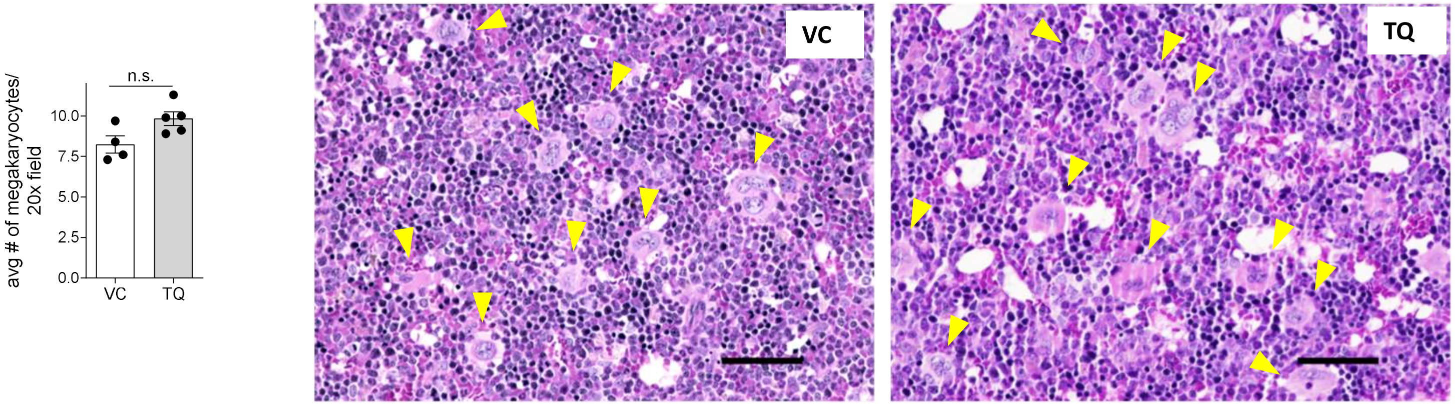

Supplementary Figure S1. MK count in the BM of tumor-free mice treated with TQ. Mice (F1 progeny of C57Bl/6 x FVB/n) were treated with TQ (30 mg/kg/day in drinking water) or vehicle control (VC) for 10 days. Femur bones were collected, slides were prepared and stained with H&E, and MKs were counted in 5-7 fields of view per bone. Average number of MKs per field of view was calculated. Left, individual data for each mouse, mean (MK count per field of view), and SEM are shown. Right, examples of staining. Magnification, 20x; scale bars, 50 µm. Arrows indicate MKs. Statistics: n.s. – non-significant in unpaired two-tailed Student's t test.
