# Supplementary material for: S100A8/S100A9 Promote Progression of Multiple Myeloma via Expansion of Megakaryocytes
Source: Cancer Res Commun. 2023 Mar 13;3(3):420–30. doi: 10.1158/2767-9764.CRC-22-0368 (PMC10010194; doi:10.1158/2767-9764.CRC-22-0368)
Supplement: Figure S2 — Expression of S100A8 and S100A9 proteins using in vivo electroporation technique. [file crc-22-0368-s03.pdf]

Supplementary Figure S2

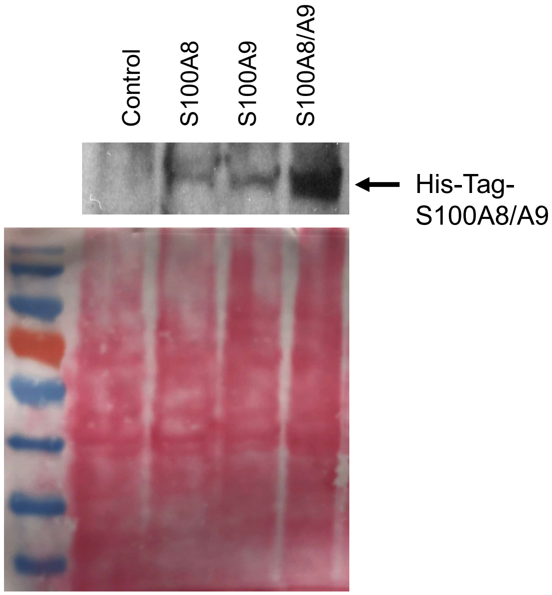

Supplementary Figure S2. Expression of S100A8 and S100A9 proteins using *in vivo* electroporation technique. Plasmid(s) encoding His-Tag and *S100a8* and/or *S100a9* were utilized to express indicated proteins in S100A9 KO mice using *in vivo* electroporation technique. In controls, mice received empty vector. Mice were euthanized on day 12 after the procedure. Expression of indicated proteins was detected in the BM using western blotting. Membranes were probed with antibody against His-tag. Loading was confirmed by Ponceau staining.
