# Supplementary material for: S100A8/S100A9 Promote Progression of Multiple Myeloma via Expansion of Megakaryocytes
Source: Cancer Res Commun. 2023 Mar 13;3(3):420–30. doi: 10.1158/2767-9764.CRC-22-0368 (PMC10010194; doi:10.1158/2767-9764.CRC-22-0368)
Supplement: Figure S3 — Effect of TPO and S100A9 on in vitro MK differentiation. [file crc-22-0368-s04.pdf]

Supplementary Figure S3

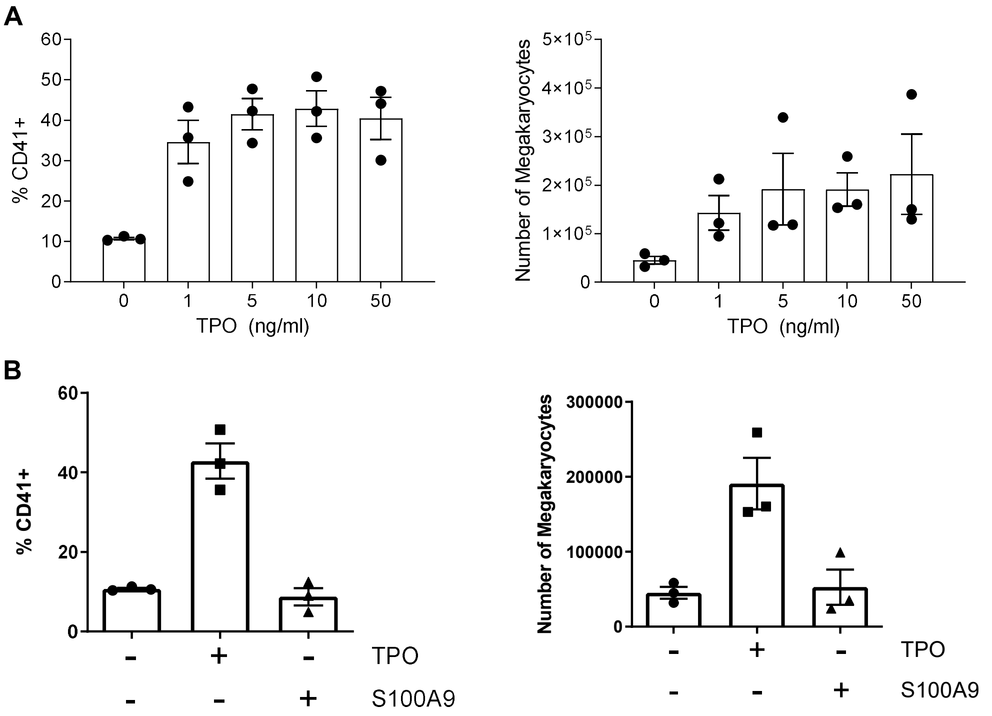

Supplementary Figure S3. Effect of TPO and S100A9 on *in vitro* MK differentiation. BM cells isolated from WT mice were cultured in the presence of indicated concentrations of TPO (**A**) or 5 µg/mL S100A9 protein (**B**). Presence and number of MK were determined on day 5 of culture. Individual values from independent experiments, mean, and SEM values are shown.
