# Supplementary material for: S100A8/S100A9 Promote Progression of Multiple Myeloma via Expansion of Megakaryocytes
Source: Cancer Res Commun. 2023 Mar 13;3(3):420–30. doi: 10.1158/2767-9764.CRC-22-0368 (PMC10010194; doi:10.1158/2767-9764.CRC-22-0368)
Supplement: Figure S4 — Effect of S100A9 protein on mouse MKs. [file crc-22-0368-s05.pdf]

**Supplementary Figure S4**

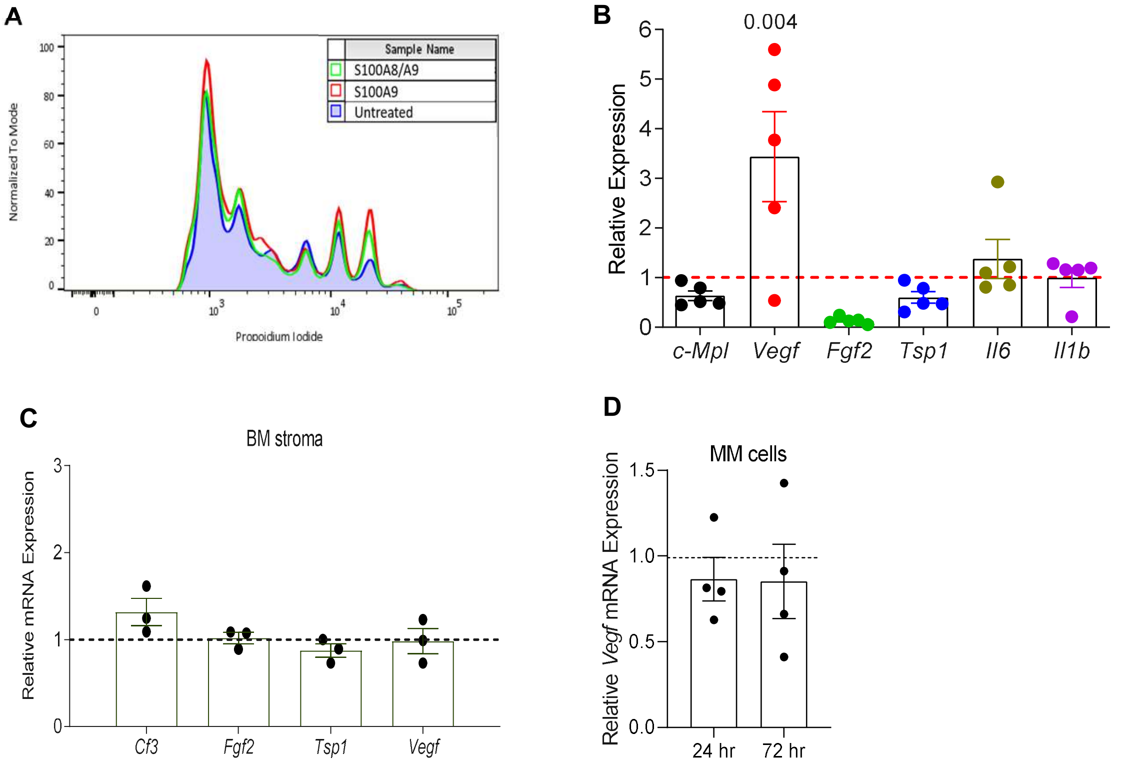


**Supplementary Figure S4. Effect of S100A9 protein on mouse MKs.** (**A**) Representative histogram demonstrating ploidy of MKs generated in the presence of S100A9 protein (5µg/mL) and TPO. (**B**) MKs were generated from mouse BM cells in the presence of TPO, collected, and cultured for 24h with or without S100A9 protein. Gene expression of indicated factors was evaluated by qPCR. Baseline level (control, without S100A9) is indicated by the dotted line. Individual values from 5 independent experiments, mean, and SEM values are show. Statistics: two-tailed Student's t test. (**C**) Gene expression of indicated factors was evaluated in stroma generated from BM cells after 24h treatment with murine S100A9. (**D**) Expression of VEGF by mouse 5TGM1 MM cells cultured in the presence of murine S100A9 protein for 24h and 72h was evaluated by qPCR. Baseline level (control, without S100A9) is indicated by the dotted line. Individual values from independent experiments, mean, and SEM values are shown.
