# Supplementary material for: S100A8/S100A9 Promote Progression of Multiple Myeloma via Expansion of Megakaryocytes
Source: Cancer Res Commun. 2023 Mar 13;3(3):420–30. doi: 10.1158/2767-9764.CRC-22-0368 (PMC10010194; doi:10.1158/2767-9764.CRC-22-0368)
Supplement: Figure S5 — BM angiogenesis in tumor-free and MM-bearing mice treated with TQ. [file crc-22-0368-s06.pdf]

Supplementary Figure S5

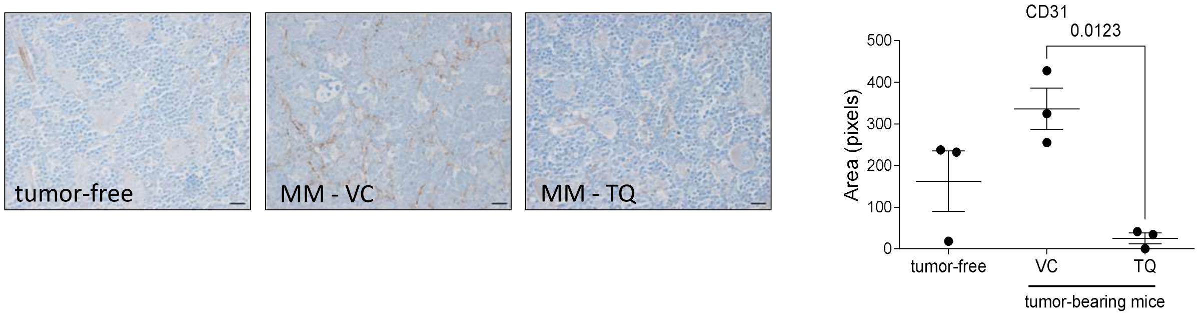

Supplementary Figure S5. BM angiogenesis in tumor-free and MM-bearing mice treated with TQ. DP42 tumors were established in syngeneic mice by i.v. injection of tumor cells. Treatment with TQ started on day 3 after tumor cell injection**.** BM angiogenesis was determined on day 19 by staining BM sections with anti-CD31 Ab. Slides were scanned and analyzed using Aperio software. Representative images (left) and quantitation (right) are shown. Scale bars, 10µm. Statistics: ANOVA with Tukey's multiple comparisons test.
