# Supplementary material for: S100A8/S100A9 Promote Progression of Multiple Myeloma via Expansion of Megakaryocytes
Source: Cancer Res Commun. 2023 Mar 13;3(3):420–30. doi: 10.1158/2767-9764.CRC-22-0368 (PMC10010194; doi:10.1158/2767-9764.CRC-22-0368)
Supplement: Figure S6 — Mechanism of S100A9 effect on MK differentiation. [file crc-22-0368-s07.pdf]

Supplementary Figure S6

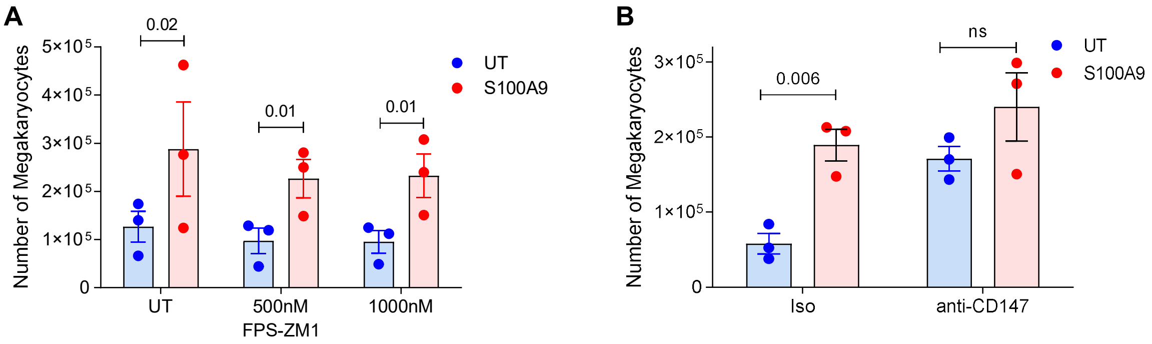

Supplementary Figure S6. Mechanism of S100A9 effect on MK differentiation. BM cells were isolated from WT mice and cultured in the presence of TPO with or without S100A9. RAGE inhibitor (**A**), anti-CD147 Ab (10µg/ml) or isotype control (**B**) were added to the cultures on day 0. Number of MKs was determined on day 5 of culture. Individual values, mean, and SEM values are shown. UT – untreated (no S100A9 added).
