# Supplementary material for: S100A8/S100A9 Promote Progression of Multiple Myeloma via Expansion of Megakaryocytes
Source: Cancer Res Commun. 2023 Mar 13;3(3):420–30. doi: 10.1158/2767-9764.CRC-22-0368 (PMC10010194; doi:10.1158/2767-9764.CRC-22-0368)
Supplement: Figure S7 — Effect of TQ in S100A9KO MM-bearing mice. [file crc-22-0368-s08.pdf]

Supplementary Figure S7

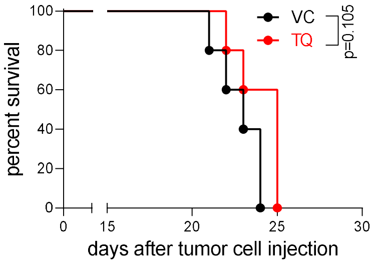

Supplementary Figure S7. Effect of TQ in S100A9KO MM-bearing mice. DP42-bearing S100A9KO mice were treated with TQ (30 mg/kg/day in drinking water, n=5) or vehicle control (n=5). Survival of mice was evaluated. Statistics: log-rank test.
