# Supplementary material for: S100A8/S100A9 Promote Progression of Multiple Myeloma via Expansion of Megakaryocytes
Source: Cancer Res Commun. 2023 Mar 13;3(3):420–30. doi: 10.1158/2767-9764.CRC-22-0368 (PMC10010194; doi:10.1158/2767-9764.CRC-22-0368)
Supplement: Figure S8 — Effect of TQ on MM cells. [file crc-22-0368-s09.pdf]

Supplementary Figure S8

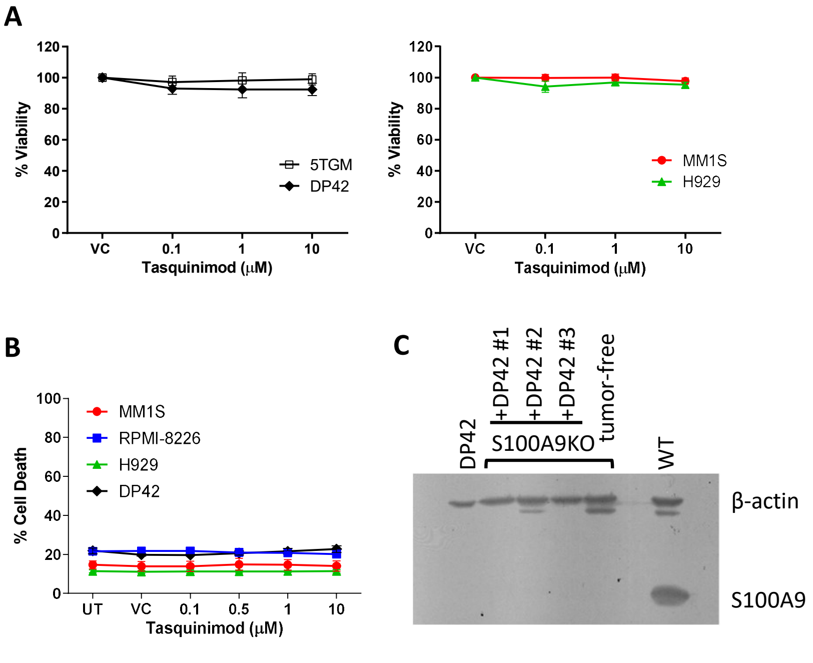

Supplementary Figure S8. Effect of TQ on MM cells. (**A**) Viability of MM cells was determined by MTT assay after 48h treatment with indicated concentrations of TQ or vehicle control (VC, DMSO). (**B**) Apoptosis of MM cells evaluated by Annexin V binding assay after 24h of treatment with TQ or VC. UT – untreated cells. Individual values, mean, and SEM values are shown. (**C**) Expression of S100A9 protein was determined by western blotting in DP42 cells cultured *in vitro* (DP42) and in BM cells from 3 individual DP42-bearing S100A9KO mice isolated 2 weeks after tumor cell injection (S100A9KO+DP42). In control, BM cells isolated from tumor-free S100A9KO mice and WT mice were used.
